# Evidence of exposure of domestic pigs to Highly Pathogenic Avian Influenza H5N1 in Nigeria

**DOI:** 10.1038/s41598-018-24371-6

**Published:** 2018-04-12

**Authors:** Clement Meseko, Anja Globig, Jeremiah Ijomanta, Tony Joannis, Chika Nwosuh, David Shamaki, Timm Harder, Donata Hoffman, Anne Pohlmann, Martin Beer, Thomas Mettenleiter, Elke Starick

**Affiliations:** 1 Regional Laboratory for Animal Influenza, National Veterinary Research Institute, Vom, Nigeria; 2 Institute of Diagnostic Virology, Friedrich-Loeffler-Institut, Insel Riems, Germany; 3 Institute of Epidemiology, Friedrich-Loeffler-Institut, Insel Riems, Germany; 4 Institute of Molecular Virology and Cell Biology, Friedrich-Loeffler-Institut, Insel Riems, Germany

## Abstract

Avian influenza viruses (AIV) potentially transmit to swine as shown by experiments, where further reassortment may contribute to the generation of pandemic strains. Associated risks of AIV inter-species transmission are greater in countries like Nigeria with recurrent epidemics of highly pathogenic AI (HPAI) in poultry and significant pig population. Analysis of 129 tracheal swab specimens collected from apparently healthy pigs at slaughterhouse during presence of HPAI virus H5N1 in poultry in Nigeria for influenza A by RT-qPCR yielded 43 positive samples. Twenty-two could be determined by clade specific RT-qPCR as belonging to the H5N1 clade 2.3.2.1c and confirmed by partial hemagglutinin (HA) sequence analysis. In addition, 500 swine sera were screened for antibodies against influenza A virus nucleoprotein and H5 HA using competition ELISAs and hemagglutination inhibition (HI) tests. Serologically, 222 (44.4%) and 42 (8.4%) sera were positive for influenza A virus NP and H5 antibodies, respectively. Sera reacted to H5N1 and A/H1N1pdm09 strains by HI suggesting exposure of the Nigerian domestic pig population to these viruses. We report for the first time in Nigeria, exposure of domestic pigs to H5N1 virus. This poses potential public health and pandemic risk due to interspecies transmission of avian and human influenza viruses.

## Introduction

Scientific investigations predict that avian influenza viruses (AIV) might adapt in swine host and contribute to the generation of a potentially pandemic strain through genetic reassortment between avian and human viruses^1^. This is due to the susceptibility of pigs to infection with avian and mammalian influenza viruses, because of possessing both avian (alpha 2,3) and mammalian (alpha 2,6) sialic acid receptors for influenza A viruses in their upper respiratory tract. Consequently, pigs could play the role of an intermediate host in the reassortment of genes through inter-species transmission like it happened prior to 2009 influenza pandemic and are therefore important in the emergence of new strains of influenza virus^2,3^. Influenza A virus (IAV) genome is composed of eight distinct RNA segments encoding at least 11 proteins which coordinate functions, components and structure of the virus. If two parent viruses co-infect a host cell, they can, through reassortment, exchange genome segments in the progenies. This reassortment of genes and further point mutations are responsible for genetic diversity that gives rise to virus adaptation in new host and emergence of novel and pandemic strains^4,5^. One of the greatest public health concerns with enzootic circulation of highly pathogenic avian influenza virus (HPAIV) in agricultural scenarios relates to exposure of humans at the human-animal interface. This bears potential to cause infection in human through direct transmission from infected poultry or through an intermediate host such as pigs^6^.

The risks involved in this inter-species transmission of influenza virus and the odd that reassortment of genes in pigs as a mixing vessel would occur is greater in countries like Nigeria with recurrent epidemics of HPAI in regions with high pigs population often in direct contact with poultry. This is because lapses in biosecurity provide ample opportunities for intra- and inter-species co-infections^7^.

Shortly after initial cases of pandemic influenza A/H1N1pdm09 reports in human worldwide, the virus was also transmitted to pigs in a reverse zoonotic fashion in many countries including Nigeria^8,9^. Nigeria has also experienced recurrent epidemics of HPAI in poultry, which underscores the likelihood of inter-species transmission of IAV in an epidemiological niche where multiple species co-mingle^7,10–12^. Therefore, the need to monitor IAV ecology and epidemiology predicated a risk-based surveillance carried out in a pig slaughter facility in Nigeria.

## Results

### Molecular characterisation and phylogenetic analyses

Apparently healthy domestic pigs from backyard and free range husbandry system in Plateau State, north central Nigeria slaughtered at the Jos central abattoir from 2015–2016 were investigated. Forty three of 129 (33%) tracheal swab specimens collected from these pigs were positive for influenza A virus (M and NP) genes by conventional and RT-qPCR assays. In order to avoid multiple freeze-thawing steps, and to save expensive RT-qPCR material, only samples with lower Cq values were used for further characterisation. Also, not all of these positive samples were investigated in the subsequent tests because of low volume of remaining RNA. Thirty positive samples were tested in multiplex RT-qPCR assays specific for HA and NA subtypes of European porcine influenza viruses. All of them were negative in the HA test (i.e. H1pdm-, H1av-, H1hu- and H3-negative), but 26 were positive for N1. Out of the 23 samples tested by the H5N1 duplex assay, 22 were positive in the H5 reaction and two in the N1 part (Table 1). In addition, these 22 samples could be determined as belonging to the H5N1 clade 2.3.2.1c by a clade-specific RT-qPCR^13^.

**Table 1:** Summary of the results of RT-qPCR tests: Cq-values of nucleoprotein gene and indicated subtypes for representative samples.

Sample ID	AIV RT-qPCR (Cq values)									
	NP^14^	HA4plex PCR^15^				NA3plex PCR^15^			Duplex PCR	
		H1pdm	H1av	H1hu	H3	N1	N2	N1-pdm	H5	N1
Sw48	35.55	neg	neg	neg	neg	33.0	neg	neg	32.06	41.09
Sw49	28.75	neg	neg	neg	neg	31.20	neg	neg	not done	not done
Sw50	33.45	neg	neg	neg	neg	35.63	neg	neg	31.13	neg
Sw53	32.77	neg	neg	neg	neg	34.79	neg	neg	32.38	neg
Sw54	33.62	neg	neg	neg	neg	33.27	neg	neg	31.31	neg
Sw55	27.56	neg	neg	neg	neg	36.40	neg	neg	31.14	neg
Sw59	34.41	neg	neg	neg	neg	35.56	neg	neg	29.94	neg
Sw60	32.94	neg	neg	neg	neg	27.40	neg	neg	29.96	29.92
Sw61	34.25	neg	neg	neg	neg	28.81	neg	neg	30.39	31.39
Sw62	33.77	neg	neg	neg	neg	31.73	neg	neg	30.42	neg
Sw65	33.99	neg	neg	neg	neg	33.67	neg	neg	31.18	neg
Sw66	34.74	neg	neg	neg	neg	34.10	neg	neg	30.81	neg
Sw67	34.70	neg	neg	neg	neg	29.27	neg	neg	30.49	neg
Sw72	32.89	neg	neg	neg	neg	34.79	neg	neg	28.35	neg
Sw73	31.51	neg	neg	neg	neg	31.72	neg	neg	28.21	neg
Sw74	33.19	neg	neg	neg	neg	32.59	neg	neg	29.81	neg
Sw75	32.11	neg	neg	neg	neg	not done	not done	neg	28.55	neg
Sw77	32.46	neg	neg	neg	neg	33.56	neg	neg	not done	not done
Sw79	34.52	neg	neg	neg	neg	33.56	neg	neg	28.57	neg
Sw80	34.08	neg	neg	neg	neg	neg	neg	neg	28.10	neg
Sw81	33.41	neg	neg	neg	neg	35.56	neg	neg	28.25	neg
Sw84	33.66	neg	neg	neg	neg	29.67	neg	neg	29.67	neg
Sw85	34.03	neg	neg	neg	neg	34.11	neg	neg	28.49	neg

Query BLAST of 5 sequences that were generated via conventional RT-PCR and Sanger sequencing of an HAII fragment (about 650 nucleotides) returned closely related viruses. This included A/Chicken/Nigeria/15VIR399-1/2015, other poultry sequences from Nigeria and from other African countries in 2015 as well as from Asian countries (all H5N1) with 99% sequence homology. As determined with the clade-specific RT-qPCR and by sequence analysis, the swine H5N1 virus genomes detected in this study clustered together and along avian H5N1 clade 2.3.2.1c.

Unfortunately, the NA gene sequencing attempts repeatedly failed even with different primer sets including conventional panNA RT-PCR, both variants^14^ and new designed and customized primers for N1 based on avian H5N1 sequences from western Africa, 2015. Attempts to isolate the virus from swabs in chicken embryonated eggs and tissues in MDCK (+) cells (cell line number 0671 of the Collection of Cell Lines in Veterinary Medicine CCLV, FLI) were also not successful.

**Figure 1:**
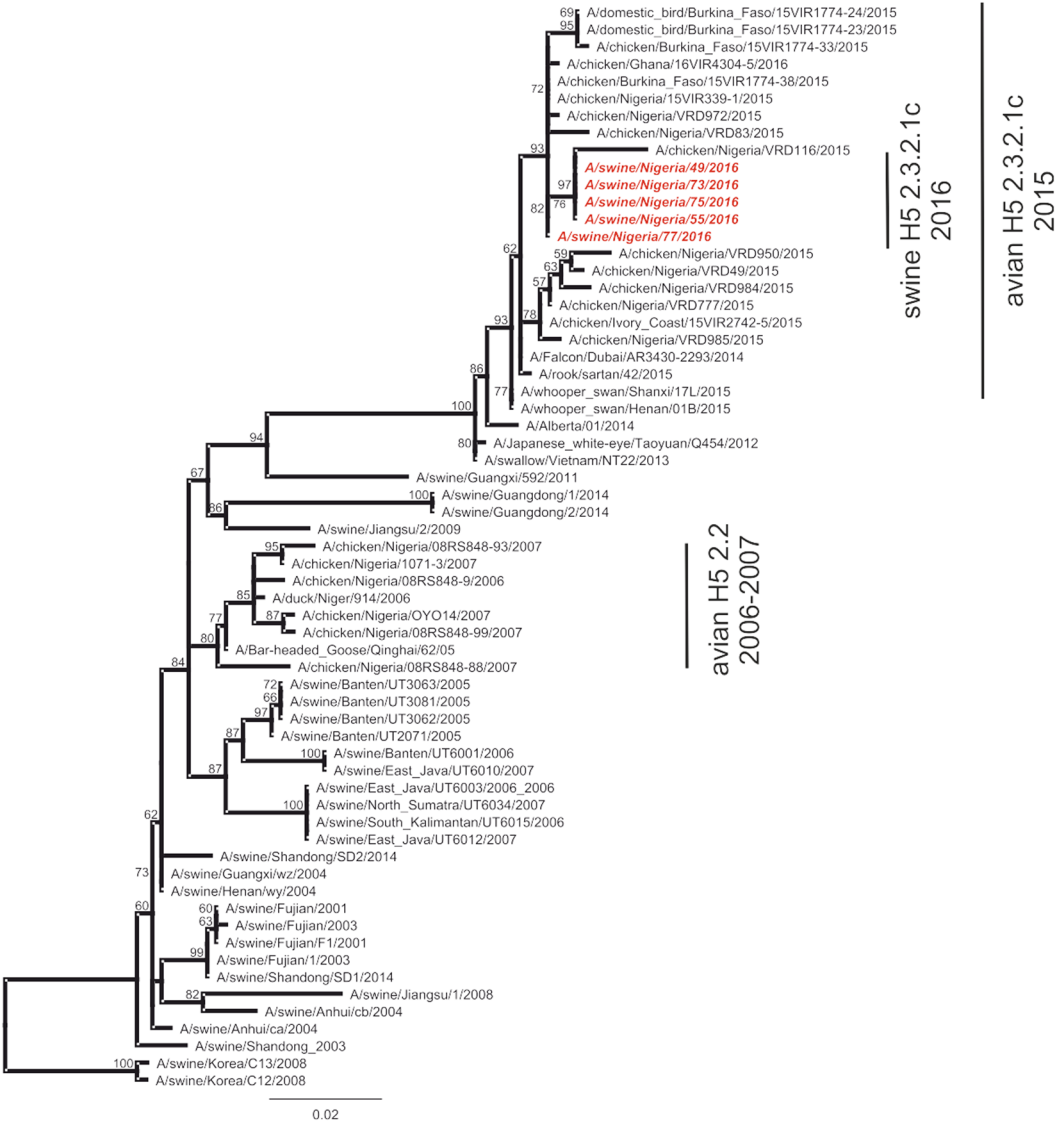
Phylogenetic tree of H5 influenza HA sequences from swine, Nigeria and avian and swine influenza sequences from GISAID and GenBank. Labels indicate the different clusters.

The analysis of the nucleotide sequences of the swine viruses showed only three silent nucleotide exchanges in the HAII gene fragment compared to A/Chicken/Nigeria/15VIR399-1/2015 H5N1. These include G, A, and C replacing A, G, and T respectively in position 1152, 1177 and 1335 of the HA gene. The partial sequences of the HA gene of the five amplifiable H5N1 has been deposited in GISAID-Epiflu^TM^ database and assigned accession number EPI262933 (A/swine/Nigeria/49/H5N1), EPI262932 (A/swine/Nigeria/55/H5N1), EPI262934 (A/swine/Nigeria/73/H5N1), EPI262935 (A/swine/Nigeria/75/H5N1), EPI262950 (A/swine/Nigeria/77/H5N1). Phylogenetically, the topology of the HA tree (Fig. 1) also clustered the virus in clade 2.3.2.1c H5N1 viruses that currently circulate in Nigeria and other West African countries.

### Serology

Five hundred swine sera were collected from apparently healthy domestic pigs also in Jos central abattoir, north central Nigeria and in Enugu slaughter slab, south east Nigeria in 2016 and 2013 respectively out of which 222 (44.4%) were positive by IDvet NP-ELISA. Among the 300 investigated serum samples from 2016, 183 (61%) reacted positive in the IDvet NP-ELISA. Of those, 42 (14%) showed positivity in the IDvet H5 ELISA. All 200 serum samples from 2013 when HPAIV was not present in poultry in Nigeria were negative in the H5-ELISA though 39 (19.5%) were NP positive. HI test against antigen for HPAI H5N1 clade 2.3.2.1.c confirmed H5 antibodies with HI titres ranging from 160 to 1280 in selected 6 of the 42 H5-ELISA positive sera from year 2016. Sera reacted also against LPAI H5N3 virus confirming that the specific component of the sera must be antibodies against H5. No cross reactivity was obtained with H5 HPAI viruses of other clades, i.e. 2.3.4.4b (H5N8) or 2.2 (H5N1). Fourteen NP-positive / H5 negative samples that were tested against swine-specific influenza virus antigens showed positive reaction predominantly for A/H1N1pdm09 with HI titres ranging from 320 to1280. Tables 2, 3 and 4 present the summary of the results of serology.

**Table 2:** Summary of NP (Nucleoprotein) and H5 influenza ELISA serology on three categories of sera collected from Jos (2013 and 2016) and Enugu (2013).

Sera collection	ELISA serology					
	NP			H5		
	total	Pos	%	total	pos	%
Jos abattoir, 2016	300	183	61	183	42	22.9 (14)
Jos abattoir, 2013	100	15	15	15	0	0
Enugu slaughter slab, 2013	100	24	24	24	0	0
Total	**500**	**222**	**44**.**4**	**222**	**42**	**18**.**9** (**8**.**4**)

**Table 3:** Sero-reactivity of swine influenza strains H1avN1av, H1huN2, H1N1pdm and H3N2 on selected NP-positive sera.

Antigens		Field sera ID (titre)													
Subtype	Virus strain	353	186	30	24	100	202	15	56	286	72	48	129	204	293
H1avN1av	A/sw/Germany/R1738/2011	80	40	160	20	80	40	20	20	160	40	160	160	40	40
H1huN2	A/sw/Germany/R2107/2010	40	20	640	40	80	10	20	80	40	40	80	20	20	20
H1N1pdm	A/sw/Germany/R26/2011	1280	1280	640	320	320	320	320	320	1280	640	1280	640	640	640
H3N2	A/sw/Germany/R96/2011	10	10	80	10	10	20	10	10	10	20	10	10	10	10

**Table 4:** Sero-reactivity of H5 strains to H5N1 2.3.2.1c, H5N8 2.3.4.4, H5N1 2.2. and H5N3 on selected NP positive sera.

Subtype/clade	Virus strain	Sera ID					
		106	192	229	294	232	242
H5N1 2.3.2.1c	A/Dubai/AR/3435/14	640	640	1280	160	320	320
H5N8 2.3.4.4.b	A/Tufted duck/AR/8444/16	10	10	10	10	10	10
H5N1 2.2.	A/Whooper swan/Germany/R65/2006	40	10	10	160	20	80
H5N3	A/Common teal/England/7894/2006	80	80	640	320	160	320

## Discussion

Infections of swine with AIV in nature seem to be rare, and only very few studies have described detection of HPAIV H5N1 in healthy domestic pigs in Indonesia and China^15,16^. In those studies, the virus appeared to have been transmitted from poultry. Our investigation revealed natural exposure of HPAIV H5N1 clade 2.3.2.1.c possibly from infected poultry in Nigeria to domestic pigs as we could find H5N1 RNA in 43 tracheal specimens of swine sampled in December, 2015 to February, 2016 in the slaughter house in Jos, Plateau state, Nigeria. Partial sequencing of the HA showed highest homology to A/Chicken/Nigeria/15VIR399-1/2015 (H5N1) with sequence homology of 99%. HPAIV H5N1 of clade 2.3.2.1c has been circulating in Nigeria since 2015 in poultry and is most prevalent in the Northern region of the country where this study was carried out^7,11,12^. In an RT-qPCR 3plex assay for the detection of swine influenza NA subtypes, it was possible to detect 22 N1-positive samples. However, sequencing of the neuraminidase failed due to the compromised quality of the samples as a result of multiple freeze thawing. Therefore, the sequence identity of the N1 could not be ascertained. Further investigation through surveillance, virus isolation and full genome sequencing is required. Another limitation was the inability to sequence the internal genes due to low quality of the remaining RNAs and low volume of original material which restricted the range of investigations including virus isolation attempts.

Significant exposure to influenza viruses was demonstrated serologically with 222 (44.4%) and 42 (8.4%) out of 500 sera samples being positive in the competitive ELISAs for the detection of NP and H5 antibodies, respectively. This was also confirmed by HI assay against H5 and H1 antigens for selected samples. Interestingly, the HI test also indicated cross reactive antibodies in low titres to other serotypes of porcine influenza viruses including H1avN1av, H1huN2, and H3N2 in the study population (Table 3a). However, the sera were almost exclusively strongly positive for the A/H1N1pdm09 antigen. The serological results also confirm exposure of swine to HPAIV H5N1 and H1N1pdm09 in Nigeria.

In a study published in 2001^17^ on co-circulation of avian H9N2 and contemporary human H3N2 viruses, possible inter-species transmission and genetic reassortment in pig was suggested. Also in 2011, influenza A H3N2 variant virus infection in a person who attended an agriculture fair in the USA was detected for the first time and characterised as a reassortant of a SIV H3N2 with A/H1N1pdm09^18^ which was further spreading among swine and humans in following years^19,20^. These reports raised concerns on the possible emergence of zoonotic influenza virus from swine as consequences of inter-species transmission^18^. Our results provide additional scientific evidence of natural exposure of HPAIV subtype H5N1 currently circulating in Nigerian poultry to pigs in the same epidemiological zone. The risk of possible gene reassortments may be due to presence of different IAV subtypes in domestic animals in the region. Furthermore, this study shows that HPAI H5N1 clade 2.3.2.1c was present in swine population in the study area as also revealed serologically through detection of H5 antibodies.

We speculate that exposure in swine is due to intensity of AIV outbreaks in the region and the breakdown in control measures. Because AIV detection in swine was not only once and not in the same group of pigs based on different sampling dates and ownership, we can also speculate that exposure was more than once within the epidemiological zone likely due to between-farms transmission similar to observations by Ssematimba *et al*.^21^. Mechanical carriage may be possible, but this does not exclude other routes of transmission. Specifically, tracheal swabs (lower respiratory tract) not nasal samples were collected. In addition 8.4% seroconversion to H5N1 in pigs was also observed in the sampled population.

In a recent experiment by Kaplan *et al*.^22^, it was observed that clade 2.3.4.4 HPAIV H5 viruses were poorly adapted for replication and transmission in pigs. In that study, inoculated pigs did not produce HI antibodies. Comparatively, our investigation showed that H5 clade 2.3.2.1c produced high antibody titre in naturally exposed pigs and corroborate observation by Kaplan *et al*.^22^, that other lineages of H5 and H1N1pdm09 are more reactive serologically in swine. Though as also observed by Balzli *et al*.^23^. AIV may produce lower respiratory tract infection in pigs based on detection of IAV by RT-qPCR in bronchoalveolar lavage fluid (BALF). However poor replication efficiency shown by high Cq values and lack of virus isolation (observed in our investigation and in the data by Kaplan *et al*.) suggests low adaptation in swine host^22^. Though domestic pigs showed low susceptibility to HPAI H5N1^24,25^, genetically engineered reassortants of H5N1 and H1N1pdm09 have been shown experimentally to have potential for mammalian transmissibility in guinea pigs^26^.

The virus RNA detected in our study were obtained from apparently healthy pigs; asymptomatic and therefore silent transmission of a potentially zoonotic HPAIV in a country with a large population of pigs in intensive and free range husbandry bears public health risks^27,28^. Thus, the large concentration of people and livestock in urban and semi-urban settings provides a veritable ground for emergence and spread of a zoonotic pathogen at the human-animal interface. This was emphasised in a previous study on the prediction and prevention of the next pandemic^29^ and also verified by serological investigations performed among Nigerian poultry workers which demonstrated H5 reactive sera within this risk group^30^. In addition, it was also observed that the emergence of zoonotic pathogens that have dominated the pandemics of the past 100 years correlates strongly with human population density and global distribution of animals^29,31^.

The significance of our study is the detection of HPAI H5N1 RNA of clade 2.3.2.1c and H5 reactive sera in apparently healthy pigs in Nigeria. Nigeria recorded the highest number of outbreaks of HPAIV in West Africa since 2006 and from 2015 to 2016. Similarly, A/H1N1pdm09 strain is dominant in domestic pigs in the region following 2009 pandemic with seroprevalence in the range of 27.4 to 29% reported in different studies^7,32,33^. If AIV is sustained and adapted in pigs, the consequences comprise suggestions by some investigators^34,35^ that a reassortant H5N1 with A/H1N1pdm09 internal genes may have enhanced replication and transmissibility competence compared to the parent H5N1. A previous serosurvey of H5N1 in pigs in Nigeria during 2006–2008 epidemic returned negative results^36^. However, the viruses that circulated in Nigeria at that time belonged to a different clade of Asian origin H5 HPAIV (clade 2.2.). Sera analysed by HI in the present investigation was negative to that clade further showing that exposure of pigs to H5 virus is a recent event. Thus detection of H5N1 in the current study suggests possibilities of enhanced factors including increased contact rates between avian and swine and widespread H5N1 virus dissemination in the environment, which could aid exposures and biological adaptation in swine host. Furthermore, a large proportion of serum samples showed antibodies against IAV most of which were reactive to A/H1N1pdm09 and therefore calls attention to the theoretical possibility of simultaneous infections with different avian and mammalian influenza A viruses in the population, the basis for reassortment of genes in pigs being a mixing vessel. This also indicates that possibly, human pandemic H1N1 infections (or porcine adapted variants thereof) play the main role for swine influenza in Nigeria. Alternatively, other swine-adapted viruses may circulate which are antigenically distinct from those of Europe or other regions.

In conclusion, the exposure to H5N1 HPAIV from avian to swine, poses yet unknown public health risk if subclinical infection in pigs is sustained and may combine with A/H1N1pdm09 also detected in the population. This might allow the generation of new potentially zoonotic viruses by gene reassortment. The potential of this IAV transmission to humans thus requires further monitoring and investigation of the swine population in Nigeria, especially in regions with high swine density and endemicity of HPAIV in poultry though the risk is up to now theoretical.

## Methods

### Sample collection

**Figure 2:**
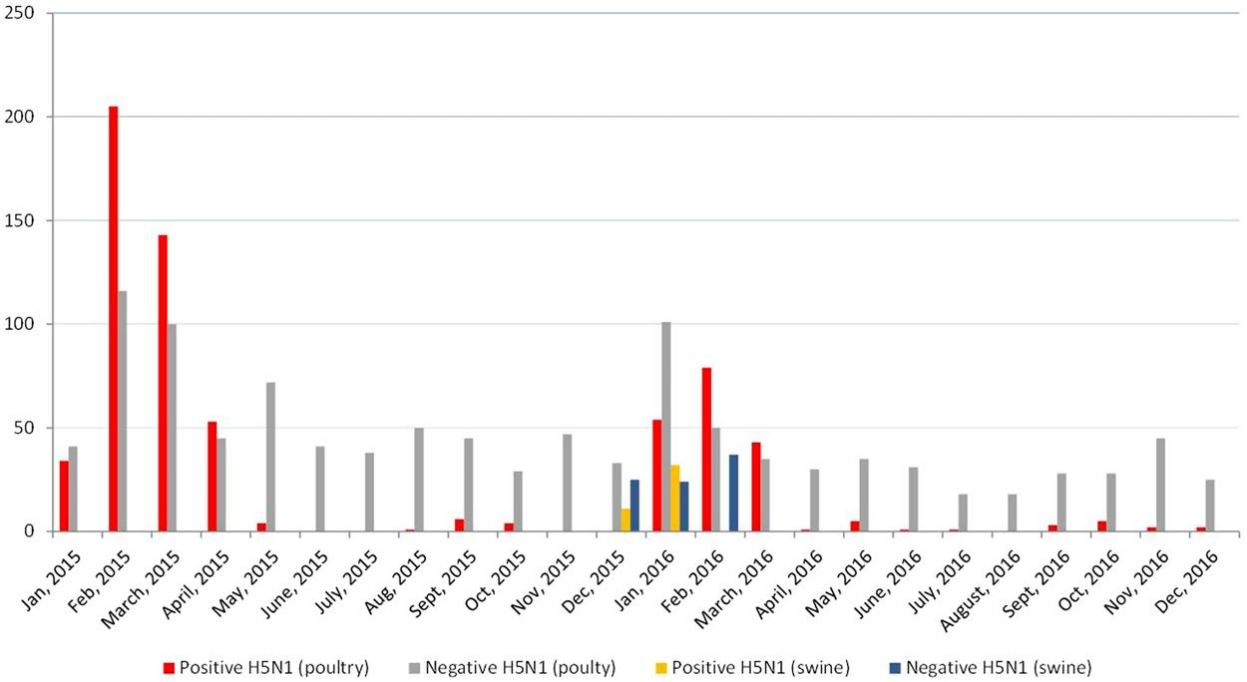
Two-year (2015–2016) temporal distribution of HPAI H5N1 in poultry in Nigeria (avian data derived from^11,12,45^) and targeted surveillance in swine from Dec 2015 to Feb 2016 showing AIV detection in swine during HPAIV outbreaks in poultry. Number of samples shown in y-axis and date of collection shown on the x-axis.

**Figure 3:**
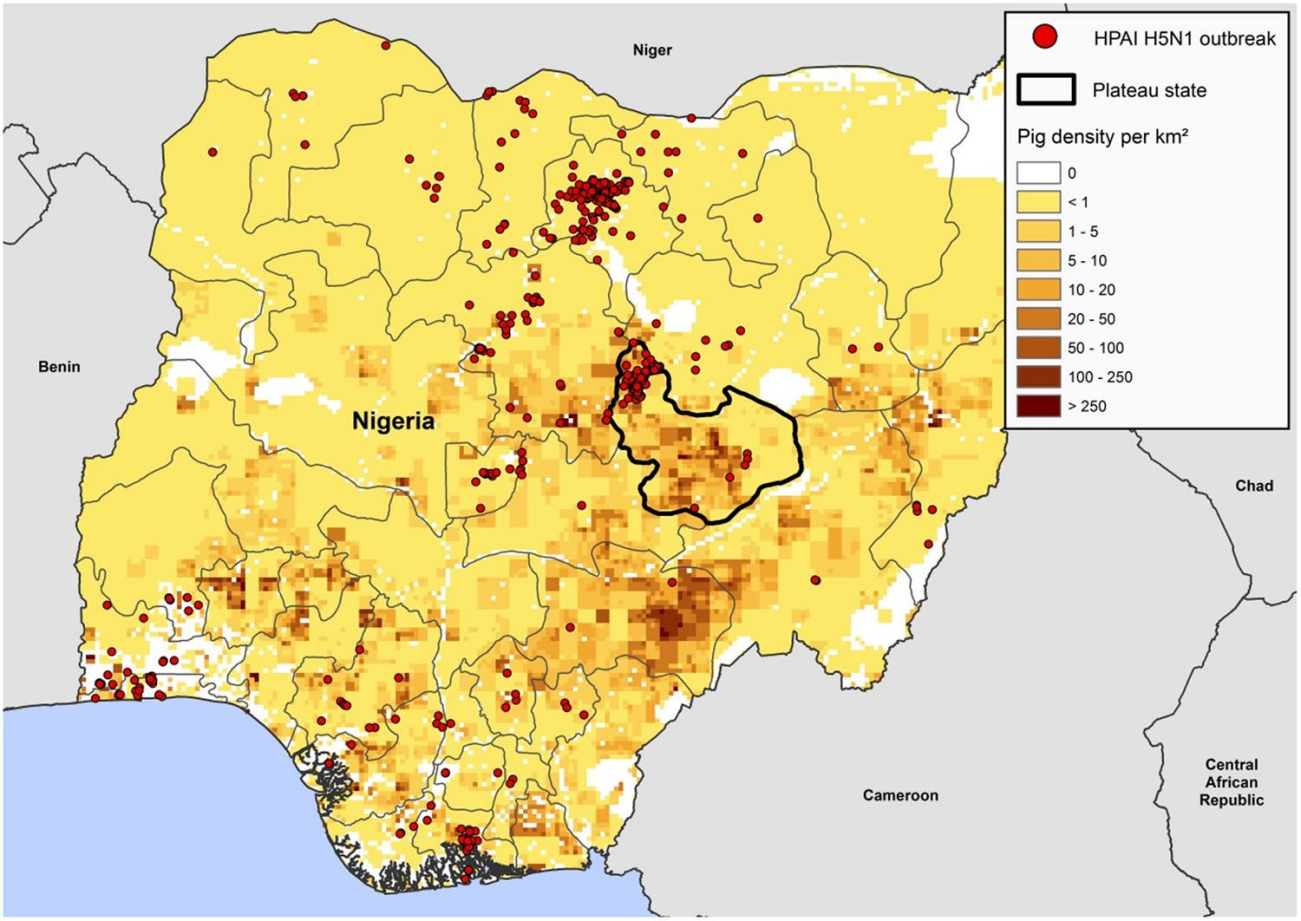
Map of Nigeria, Plateau state, source of pigs to the slaughterhouse. Colour bar shows density of pigs in the state while dots indicate spatial distribution of HPAI H5N1 outbreaks in poultry in 2015–2016^11,12,45^. Map generated with ArcGIS (Software: ArcGIS 10.3 by ESRI- https://www.esri.de/support-de/produkte/arcgis-for-desktop-10-3). Data source: Global Administrative Areas (http://www.gadm.org/)^46^ and Global Pig Density (2005) – GeoNetwork by FAO (http://www.fao.org/geonetwork/srv/en/main.home).

In the study, 129 tracheal swabs and 129 lung tissues were concurrently and randomly collected through convenience sampling from apparently healthy pigs at a slaughter house in December 2015 through February 2016 in the period of avian influenza epizootic in Nigeria (Figs 2 and 3). Different owners (pig farmer/trader) brought 3 to 10 pigs in one batch to the abattoir for contract slaughter during each slaughtering day. Each batch is followed in the slaughtering line and 3 tracheal and 3 lung samples (sometimes less) were collected from each batch/farm/trader. Sampling limitation included inability to collect specimens from every batch due to owners’ resistance on account of perceived lack of compensation in the event of a positive case. This procedure was carried out on a weekly basis. These animals originated from free range and backyard pig farms in Plateau State, North central Nigeria (Fig. 3). Animals were transported from backyard and free range pig holdings in the region to the central city abattoir where only cattle, small ruminants and pigs were slaughtered in separate lines. The central city abattoir is located on approximate 400 × 200 square metre land area. The build-up facility covers up to 200 × 100 square metre and the pig slaughter section is about 80 × 15 square metre. The abattoir handles up to 70% of bred pigs in Plateau state and it serves as the major outlet for pork in the region. In the same longitudinal study 300 sera were obtained from slaughtered pigs in the facility over a period of six months from February to July 2016. Two hundred sera collected in 2013 from the same abattoir and in Enugu (southern Nigeria) were also included in the investigations for comparison. Pre-extracted nucleic acid, swabs and tissues were stored at −80 °C while sera were stored at −20 °C until they were all shipped on dry ice for analyses at the OIE/FAO and National reference laboratory for avian influenza, Friedrich-Loeffler-Institute (FLI), Isle of Riems in Germany.

### Molecular characterisation

The swab specimens were initially screened by conventional reverse transcription (RT-) polymerase chain reaction (PCR) for a conserved part of the matrix (M) gene coding region in Nigeria according to the method described elsewhere^37^. RT real time PCR (RT-qPCR) targeting parts of the nucleoprotein gene were additionally done at FLI, Germany as described by Fereidouni *et al*.^38^. Positive samples were thereafter analysed using Ambion-AgPath ID One Step RT-PCR Kit and RT-qPCR protocols for a swine influenza virus (SIV) hemagglutinin (HA) 4plex assay targeting H1pdm, H1avian (H1av), H1human (H1hu) and H3 and for a SIV-neuraminidase (NA) 3plex assay targeting N1, N2, N1pdm^14^. In addition, an in-house IAV-H5N1 duplex RT-qPCR specific for HAs and NAs of HPAIV H5N1 viruses was carried out (Friedrich-Loeffler-Institut, Germany). To confirm the high pathogenicity of the viruses in the specimen and to detect the clade of the viruses, RT-qPCR assays a) with a probe specific for HPAI virus HA cleavage sites and b) for the differentiation of 3 H5N1 clades (2.2.1.2., 2.3.2.1., 2.3.4.4.) were also performed^13^.

### Sequencing and phylogenetic analyses

Amplification was performed for highly conserved parts of HA and NA segments via conventional RT-PCR assays^39,40^. Primers used for HA - GGA ATG ATH GAY GGN TGG TAT GG-HA-(HA-1134-F) and ATA TCG TCT CGT ATT AGT AGA AAC AAG GGT GTT TT-(Bm-NS-890R) targeted a region encoding parts of the HA stalk^17^. Primers used for NA - (Mix A- ATC GAR GAR TCN TGY – Na-(886.1-F). GCA GTA TAT CGC TTG ACA AGT AGA AAC AAG G-Na (-647-R). Mix B- GTC GAR GAR TGY TCH TGB TA (NA-886.3-F), GCA GTA TAT CGC TTG ACA AGT AGA AAC AAG G (NA-647-R) were described by Gall *et al*.^40^. Amplicons were gel purified and sequenced by Sanger sequencing using the BigDye Terminator v1.1 Cycle Sequncing Kit (supplier: Applied Biosystems) at a capillary sequencer (3130x Genetic Analyser, Applied Biosystems) as previously described^41^. Sequence adjustment were done with Geneious software suite (v. 10.0.9; Biomatters, Auckland, New Zealand) to generate consensus sequences. Alignments were done using MAFFT (vs7.308)^42^, alignments were trimmed and realigned (FFT-NS-i x 1000 refinement scoring 200PAM /k = 2). Phylogenetic analysis was done with RAxML^43^ (GTR GAMMA Model + Rapid bootstrapping 1000 replicates). Query BLAST of the HA sequences in GISAID-EpifluTM (https://platform.gisaid.org/epi3/froned#365177) and NCBI Nucleotide BLAST (https://blast.ncbi.nlm.nih.gov/Blast.cgi?PROGRAM9) was done with sequences from West Africa and sequences of H5N1 obtained from pigs mostly of Asian origin.

### Serology

To assess levels and extent of exposure to influenza viruses in domestic pigs in the study area, 300 recently collected sera (2016) along with 200 archived sera (2013) were analysed for Influenza A, NP and H5 specific antibodies, respectively, using “ID-Screen” Competition ELISA assays obtained from IDVet according to manufacturer’s instructions. A subset of ELISA positive sera were subtyped by hemagglutination inhibition (HI) assay^44^ against selected reference influenza antigens of European porcine influenza viruses [H1avN1av (A/sw/Germany/R1738/2011); H1huN2 (A/sw/Germany/R1207/2010); H1N1pdm (A/Germany/R26/2011) and H3N2 (A/sw/Germany/R96/2011)] and against avian influenza viruses [H5N1 clade 2.3.2.1c (A/Falcon/Dubai/AR3435/14); H5N8 clade 2.3.4.4b (A/Tufted duck/Germany/AR8444/16); H5N1 clade 2.2 (A/Whooper swan/Germany/R65/2006); H5N3 (A/Common teal/England/7894/2006), the last one kindly provided by the European Reference Laboratory for Avian Influenza. All laboratory analyses were carried out under standard bio-containment requirement according to level of risks involved and working under biosafety cabinets in BSL-2 and 3 facilities.

The datasets generated and/or analysed during the current study are available in the GISAID-EpiFlu^TM^ - www.platform.gisaid.org/ with the following accession numbers: EPI262932, EPI262933, EPI262934, EPI262935, EPI262950.
